# Effect of Solid-State Fermentation on Vitamin C, Photosynthetic Pigments and Sugars in Willow Herb (*Chamerion angustifolium* (L.) Holub) Leaves

**DOI:** 10.3390/plants11233300

**Published:** 2022-11-29

**Authors:** Nijole Vaitkeviciene, Elvyra Jariene, Jurgita Kulaitiene, Marius Lasinskas, Ausra Blinstrubiene, Ewelina Hallmann

**Affiliations:** 1 Department of Plant Biology and Food Sciences, Agriculture Academy, Vytautas Magnus University, Donelaicio St. 58, 44248 Kaunas, Lithuania; 2 Department of Functional and Organic Food, Institute of Human Nutrition Sciences, Warsaw University of Life Sciences, Nowoursynowska 15c, 02-776 Warsaw, Poland

**Keywords:** pigments, solid-state fermentation, willow herbs, sugars

## Abstract

The goal of this investigation was to establish the impact of solid-state fermentation of different durations on the quantitative changes of vitamin C, sugars and photosynthetic pigments in the leaves of willow herbs. The tested leaves were fermented using two solid-state fermentation methods (aerobic and anaerobic) for different time periods (unfermented and fermented for 24, 48 and 72 h). The quantitative and qualitative composition of chlorophylls, carotenoids, sugars and vitamin C were determined using high performance liquid chromatography (HPLC) with UV detectors. Results indicated that aerobic and anaerobic solid-state fermentation significantly decreased the contents of vitamin C, dehydroascorbic and L-ascorbic acids in leaves compared with the unfermented leaves. The contents of total chlorophyll and chlorophyll a were the highest in unfermented leaves and after 24 h of aerobic solid-state fermentation. The maximum content of total carotenoids in leaves were after 48 and 72 h of aerobic solid-state fermentation (149.31 mg 100 g^−1^ and 151.51 mg 100 g^−1^, respectively). The application of anaerobic solid-state fermentation resulted in significant increase in the content of total sugars, fructose and glucose in investigated samples. In conclusion, optimization of fermentation parameters allows increasing the content of sugars and photosynthetic pigments in leaves of willow herbs.

## 1. Introduction

Willow herb (*Chamerion angustifolium* (L.) Holub) is an important medicinal plant, which is common to most areas of Asia, Europe, and North America. The plant belongs to the Onagraceae family [1]. It has long been used in folk medicine as an analgesic, sedative, astringent, anti-inflammatory, antimicrobial, antisclerotic and diaphoretic agent. These characteristics are the result of the availability of many groups of bioactive substances [2,3]. Nowadays, it is also used in food, pharmaceutical, and cosmetic industries [4]. This plant is rather easy to cultivate and quickly accumulates a high content of biomass, which makes it a potential source of nutrients and phytochemicals, also a valuable raw material for further processing [5]. According to the literature, leaves of *C. angustifolium* contain approximately 250 different metabolites. They are rich source of not only phenolic compounds (phenolic acids, tannins, and flavonoids) [6,7] but also some vitamins, sugars and photosynthetic pigments such as carotenoids and chlorophyll [8,9]. Various organs of willow herbs are rich in vitamin C [8]. This essential dietary component helps strengthen immune system and is necessary for the growth, development and repair of all body tissues [10]. Many diseases, such as infections, cancer, cardiovascular disease, stroke, diabetes, and sepsis, have been associated with poor vitamin C levels [11,12]. Decreased vitamin C levels in disease are often explained by a combination of a sometimes massively increased turnover due to oxidative stress and inflammation and a decreased dietary intake of vitamin C associated with the disease [13].

Other essential substances in leaves are the assimilation pigments such as chlorophylls and carotenoids, which may also increase the antioxidant characteristics [14]. According to the literature, the main soluble carbohydrates in leaves of willow herbs are fructose, glucose and sucrose [9].

Many studies showed that *C. angustifolium* exhibits significant phytochemical variability in relation to the plant part, geographical origin, time of harvest. In addition, the chemical compositions of willow herb leaves largely depend on their preparation such as fermentation [4,6,15]. Fermentation is a biotechnological process which can modify and improve nutritional value and organoleptic (color, odour, taste) characteristics of plant raw materials. Recently, fermented products have been receiving increased interest from researchers and consumers [16,17]. Therefore, it is important to choose optimal fermentation conditions for improving nutritional value and organoleptic attributes of leaves of willow herbs.

However, there is a lack of available literature on effect of different fermentation conditions on contents of chlorophylls, carotenoids, sugars and vitamin C in leaves of willow herbs. Therefore, the purpose of this investigation was to evaluate the impact of solid-state fermentation (aerobic and anaerobic) of different durations on the quantitative and qualitative composition of vitamin C, carotenoids, chlorophylls, and sugars in willow herb leaves.

## 2. Results and Discussion

### 2.1. The Contents of Vitamin C, Dehydroascorbic Acid (DHA) and L-Ascorbic Acid (LAA) 

During the solid-state fermentation of willow herb leaves, various chemical reactions occur, during which complex compounds are broken down into simpler ones, some substances are transformed into others, and thus both qualitative and quantitative changes occur [18,19,20].

From the presented data (Table 1), we can see that solid-state fermentation significantly decreased the amounts of vitamin C in its oxidized form, dehydroascorbic acid, and L-ascorbic acid. The contents of vitamin C were the highest in unfermented leaves samples (658.06 mg 100 g^−1^). The contents of this vitamin significantly decreased after aerobic and anaerobic solid-state fermentation compared to control. These results can be explained by the fact that vitamin C is very sensitive to atmospheric conditions (heat, light, air); therefore, it can be destroyed during plant raw material processing. As we can see from the obtained data (Table 1), the decrease in vitamin C content also was significantly influenced by the duration of fermentation: the longer the duration of the fermentation, the less vitamin C remained in the leaves, especially under aerobic conditions. For example, after 24 h aerobic solid-state fermentation, vitamin C decreased by 37.12% (413.77 mg 100 g^−1^ DW), after 48 h aerobic solid-state fermentation decreased by 72.81% (to 178.94 mg 100 g^−1^ DW), and after 72 h aerobic solid-state fermentation decreased by 77.87% (to 145.61 mg 100 g^−1^ DW) compared to the control (658.06 mg 100 g^−1^ DW). The greatest contents of DHA (495.22 mg 100 g^−1^) and LAA (162.83 mg 100 g^−1^) were established in not-fermented leaves. In leaves fermented under aerobic (after 24, 48 and 72 h) and anaerobic (after 24, 48 and 72 h) fermentation conditions, the contents of DHA and LAA significantly decreased compared to control.

### 2.2. The Contents of Photosynthetic Pigments

Fermentation methods and their duration produced a significant effect on quantitative changes in photosynthetic pigments (Table 2 and Table 3). 

In this experiment, we determined that the highest amounts of total carotenoids were observed after 48 and 72 h aerobic fermentation (149.31 mg 100 g^−1^ and 151.51 mg 100 g^−1^, respectively), while the lowest—after 24 h (108.83 mg 100 g^−1^ DW) (Table 2). Carotenoids are found in many plant products [21]. Cutting and pressing during solid-state fermentation could intensify cell wall degradation, thus improving diffusion of biologically active substances from the inner cell parts, and then initiating better extraction. However, the solid-state fermentation method should be selected in such a way as to allow the extraction of the carotenoids or other selected compounds.

Carotenoid lutein is structurally similar to zeaxanthin. In addition, lutein and zeaxanthin can be interconverted in the body through an intermediate called meso-zeaxanthin [22]. In aerobic and anaerobic conditions, the longer duration (48 h and 72 h) produced significantly higher values of zeaxanthin of willow herb leaves compared with not-fermented leaves (control). However, the greatest content of lutein was established in not-fermented leaves (30.61 mg 100 g^−1^ DW). The content of this carotenoid in willow herb leaves significantly decreased after 24, 48 and 72 h aerobic (51.35%, 30.87% and 32.67%, respectively) and after 24, 48 and 72 h anaerobic (58.44%, 56.84% and 50.70%, respectively) solid-state fermentation compared to control leaves. 

The highest contents of beta-carotene were established in control leaves and after 72 h under aerobic solid-state fermentation, also after 48 h and 72 h under anaerobic solid-state fermentation (7.57 mg 100 g^−1^, 7.53 mg 100 g^−1^, 7.51 mg 100 g^−1^ and 7.54 mg 100 g^−1^, respectively), but without significant differences between them.

Beta-carotene, lutein, zeaxanthin—all of these carotenoids are derivatives of tetraterpenes. The carotenoid precursor geranyl-geranyl diphosphate (GGDP) can be converted to carotenes or xanthophylls by different steps in carotenoid biosynthesis [23]. The last two steps involving (e)-4-hydroxy-3-methylbut-2-en-1-yl diphosphate synthase and reductase can only take place in a completely anaerobic environment [24]. Therefore, it is thought that this may have contributed to significantly higher levels of zeaxanthin detected in leaves using an anaerobic solid-state fermentation process compared to not-fermented leaves (Table 2). 

Primary metabolites such as chlorophylls in plants are directly involved in plant growth and development, thus they are very important for many biochemical processes: respiration, photosynthesis, and nutrient uptake [25]. Chlorophylls perform a variety of plant vital functions. In chloroplasts, chlorophylls are the predominant light-absorbing pigments involved in photosynthesis [26]. Liu and co-authors [27] found that the dominant pigment in the leaves of many plants, chlorophyll, has a variety of biological properties such as antioxidant, anti-inflammatory, and anti-cancer. 

This study showed that during the solid-state fermentation, the metabolism of chlorophylls occurs. This can be seen from the change in color intensity: the leaves of the control variant were bright green, and during the solid-state fermentation process, this color gradually changed to brownish-green until after 72 h of fermentation it became almost brown. The contents of total chlorophylls and chlorophyll a were the highest in not-fermented leaf samples (371.47 and 278.70 mg 100 g^−1^, respectively) and after 24 h of aerobic solid-state fermentation (321.36 and 281.73 mg 100 g^−1^, respectively) (Table 3). The contents of the above-mentioned components significantly decreased after 48 h and 72 h of aerobic solid-state fermentation and after 24 h, 48 h and 72 h of anaerobic solid-state fermentation compared to control, whereas the opposite trend was established for chlorophyll b in the investigated samples. The contents of this pigment in tested leaves significantly increased after 48 h and 72 h aerobic (16.91% and 26.44%, respectively) and after 24, 48 and 72 h anaerobic (3.28%, 26.44% and 44.48%, respectively) solid-state fermentation compared to control leaves. 

Moreover, during fermentation, chlorophyll compounds and other pigments undergo degradation due to the accumulation of lactic acid and the resultant decline in pH [28]. 

Based on the obtained research data, it can be stated that different conditions of solid-state fermentation (aerobic and anaerobic) and different fermentation times (24, 48 and 72 h) influenced the changes in the content of carotenoids and chlorophylls in willow herb leaves. Researchers Couto and Sanroman [20] state that the quality of the solid-state fermentation process may be affected not only by the duration of fermentation but also by other microorganisms (lactic acid bacteria (Lactobacillus plantarum) and yeast).

### 2.3. The Contents of Sugars 

Sugars are the major energy source [29]. During solid-state fermentation, one of the steps is the hydrolysis of primary polymeric substances to finer substances (e.g., disaccharides (sucrose) to monosaccharides (fructose and glucose)). 

The results of this study showed that application of anaerobic solid-state fermentation resulted in a significant increase in the content of total sugars in willow herb leaves (after 24 h—10.06%, after 48 h—27.37%, and after 72 h—23.46%) compared with the control (Table 4). However, the total sugar content significantly decreased under aerobic solid-state fermentation. This could be explained by the fact that under solid-state fermentation, the decomposition of disaccharide sucrose into monosaccharides occurs. In this case, fructose and glucose is produced more intensively under anaerobic conditions than during aerobic fermentation (Table 4). A similar trend was established for fructose and glucose in investigated samples. The maximum content of these sugars in willow herb leaves was after 48 h and 72 h of anaerobic solid-state fermentation, while the lowest—after 72 h of aerobic solid-state fermentation. The content of sucrose was, notably, the highest in control sample. However, the contents of this sugar significantly decreased after 24, 48 and 72 h aerobic solid-state fermentation (69.81%, 70.44% and 71.38%, respectively) and after 24, 48 and 72 h anaerobic solid-state fermentation (49.68%, 53.77% and 54.72%, respectively) compared to not-fermented leaves.

### 2.4. Principal Component Analysis (PCA)

**Figure 1:**
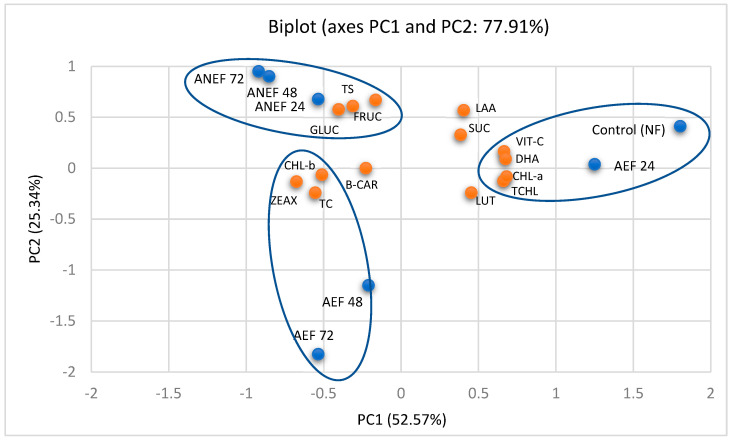
PCA results of contents of investigated compounds in willow herb leaf samples (investigated compounds: vitamin C (VIT-C), dehydroascorbic acid (DHA), L-ascorbic acid (LAA), total carotenoids (TC), zeaxanthin (ZEAX), lutein (LUT), beta-carotene (B-CAR), total chlorophyll (TCHL), chlorophyll a (CHL-a), chlorophyll b (CHL-b), total sugars (TS), fructose (FRUC), glucose (GLUC), sucrose (SUC); willow herb leaf samples: Control not-fermented (Control (NF)), aerobic solid-state fermentation 24 h (AEF 24), aerobic solid-state fermentation 48 h (AEF 48), aerobic solid-state fermentation 72 h (AEF 72), anaerobic solid-state fermentation 24 h (ANEF 24), anaerobic solid-state fermentation (ANEF 48), anaerobic solid-state fermentation 72 h (ANEF 72).

The relationships among the contents of vitamin C, photosynthetic pigments and sugars in willow herb leaves samples were determined using PCA. The PCA biplot graphically summarized the allocation of data for the investigated compounds supplied in Table 1, Table 2, Table 3 and Table 4. According to the results of PCA, two principal components (with an eigenvalue higher than 1) represent most of the information on the measured compounds, which explains 77.91% of the total variance (PC2 accounts for 52.57% of the total variance, while PC2—25.34% of it) (Figure 1).

On the principal component analysis map, the samples of willow herb leaves were classified into three groups, as shown in Figure 1. The control and AEF 24 samples were associated with higher contents of vitamin C, dehydroascorbic acid, total chlorophylls and chlorophyll a. Another group was formed by ANEF 24, ANEF 48, and ANEF 72. Due to the increased concentrations of total sugars, fructose, and glucose, this group was formed. AEF 48 and AEF 72 were grouped together due to possessing greater contents of total carotenoids and zeaxanthin.

## 3. Materials and Methods

### 3.1. Object of Research 

The leaves of willow herb (*Chamerion angustifolium* (L.) Holub) for the experiment were collected from a naturally growing willow herb habitat located in Giedres Nacevicienes ecological farm (No. SER-T-19-00910), which is in Safarka village (55°00′22″ N; 24°12′22″ E), Jonava district (Lithuania) in 2020. The total plot area of the experiment (natural habitat) was 12 ares (1200 m^2^).

### 3.2. Plant Material Preparation

The leaves of willow herbs were randomly collected from different parts of the experimental field at the stage of full flowering, in early July (I decade). For the raw material collection studies, the experiment was performed in four replicates. The variants in the repetition were randomized. Plant protection products against diseases and pests have not been used. In each replicate, 50 plants were selected for the study. From each plant, 10 to 15 leaves were collected. The leaves were collected during the day at about noon when they were dry. The composite sample of the leaves was 6.3 kg and divided for further experiment:

1. Control: 0.900 kg not-fermented (0 h).
2. Aerobic: 2.7 kg for solid-state fermentation lasting 24, 48, and 72 h.
3. Anaerobic: 2.7 kg for solid-state fermentation lasting 24, 48, and 72 h.

The process of solid-state fermentation: fresh leaves of willow herbs were carefully crushed and cut with special plastic knives. Samples of willow herb leaves were divided into 0.300 kg for each of three replications. For anaerobic solid-state fermentation, the leaves were rigidly crushed in glass containers (150 mL capacity) and covered with a lid, and for aerobic solid-state fermentation, the leaves were rigidly crushed in glass containers (150 mL capacity) and covered with an air-passing lid. The solid-state fermentation process took place at 24, 48 and 72 h in a controlled growth chamber (Sanyo, Growth Cabinet MLR-350, Japan) (Laboratory of Agrobiotechnology, Joint Research Center) at a temperature of 30 ± 0.5 °C. Control and fermented leaf samples were dried at 40 °C for 10 h using Termaks drying oven (Bergen, Norway). Then, all samples were ground with a powder grinder Grindomix GM 200 mill (Retsch GmbH, Haan, Germany) and stored in closed packages at 25 °C in a dry, dark, well-ventilated room. All biochemical leaf analyses were performed in triplicate. 

### 3.3. Methods of Laboratory Research and Analyses

The chemical composition of the leaves of willow herbs were analyzed in the Laboratory of Biochemical Re-search at the Warsaw University of Life Sciences (Poland). Separation and identification of vitamin C, carotenoids, chlorophylls, and sugar were performed in the dry weight (DW).

#### 3.3.1. Vitamin C determination 

The vitamin C amount of dried willow herb leaves was determined using an HPLC coupled to a spectrometer UV-VIS (Shimadzu Manufacturing Inc., Tampa, FL, USA) [30]. An amount of 100 mg of dried samples was weighed in a plastic tube, mixed with 5 mL of 5% meta-phosphoric acid, and shaken. Then, samples were extracted at a temperature 30 °C for 10 min in an ultrasonic bath (5.5 kHz) and centrifuged at 6000 rpm for 10 min at 0 °C. Next, 100 µL supernatant was transferred into a clear HPLC-vial. The following parameters were selected for this analysis: mobile phase acetic buffer (pH 4.4) prepared from two solutions, 63% sodium acetate (0.1 M) and 37% acetic acid (0.1 M, glacial, 99.9% purity). Isocratic flow was used with 1 mL min^−1^. Dehydroascorbic and L-ascorbic acids were identified based on the standards and their retention times. The results were expressed in mg 100 g^−1^ DW.

#### 3.3.2. Sugar Determination

The sugars of dried willow herb leaves were determined using an HPLC coupled to a spectrometer UV-VIS (Shimadzu Manufacturing Inc., Tampa, FL, USA) according to the method as described by Ponder and Hallmann [30]. An amount of 100 mg of dried samples was weighed in a plastic tube, mixed with 5 mL of 80% acetone, and stirred carefully using vortexing. Then, willow herb samples were extracted at a temperature 30 °C for 10 min in an ultrasonic bath (5.5 kHz) and centrifuged at 6000 rpm for 10 min at 3 °C. An amount of 1000 µL of clear supernatant was transferred into an HPLC vial. Under isocratic conditions, the sugar compounds were separated at a flow rate of 1 mL min^−1^ while employing 80% acetone and deionized water. The total time of analysis was 15 min. The sucrose, glucose and fructose were identified based on the standards (99.9% purity) and their retention times. The results were expressed in mg 100 g^−1^ DW.

#### 3.3.3. Chlorophylls Determination 

Chlorophyll (a and b) amounts of dried willow herb leaves were established by the HPLC (Polish agent Shimpol, Warsaw, Poland) method. This method was described by Ponder and Hallmann in 2020 [30]. An amount of 100 mg of leaf sample was dissolved in 100% acetone and extracted using an ultrasonic laboratory bath (5.5 kHz) at a temperature 0 °C for 10 min. In the next step, extracts were centrifuged at 6000 rpm for 10 min, at the temperature of 0 °C. An amount of 1 mL of extract was transferred into an HPLC vial. The wavelength for determination of chlorophylls was 445–450 nm. The amounts of individual chlorophylls were calculated using standard curves and the sample dilution coefficient. The results were expressed in mg 100 g^−1^ DW.

#### 3.3.4. Carotenoid Determination 

The qualitative and quantitative compositions of carotenoids of dried willow herb leaves were established using a method as described by Hallmann et al. [31]. An amount of 100 mg of dried leaf sample was mixed with 1 mg of magnesium carbonate and 5 mL of pure acetone, and stirred carefully with a vortex. Then, solutions were extracted using an ultrasonic laboratory bath at a temperature of 0 °C for 10 min and centrifuged (3780× *g*) at a temperature of 5 °C for 10 min. HPLC system (Shimadzu, USA Manufacturing Inc., Canby, OR, USA) was used for determination of these pigments. An amount of 800 µL of extract was transferred into an HPLC vial. Isocratic flow was used with 1 mL min^−1^, total time of analysis—28 min. The wavelength for determination of carotenoids was 445–450 nm. Zeaxanthin, lutein and beta-carotene were identified based on the external standards (Fluka and Sigma-Aldrich, Poznan, Poland). The results were expressed in mg 100 g^−1^ DW.

### 3.4. Mathematical Statistical Evaluation of Research Data

Using the STATISTIKA software program (Statistica 10; StatSoft, Inc., Tulsa, OK, USA), two-way analysis of variance (ANOVA) was used to statistically process all data. The statistical significance of differences between the means was estimated by Fisher’s least significant difference (LSD) test (*p* < 0.05). Principal component analysis was carried out to classify the willow herb leaf samples according to their chemical composition, including vitamin C, photosynthetic pigments, and sugars (using Software XLSTAT, 2018, New York, NY, USA).

## 4. Conclusions

The discussed results showed that solid-state fermentation affected the amounts of biologically active substances in the leaves of willow herbs. Vitamin C levels were significantly reduced in all variants, so it would be appropriate to recommend not-fermented willow herb leaves (control variant) as a plant source of vitamin C.

Total carotenoid content increased significantly after 48 and 72 h of aerobic fermentation, but zeaxanthin and beta-carotene levels were significantly higher after anaerobic fermentation compared to control. Therefore, according to these data, it would be possible to recommend willow herb leaves after 72 h of anaerobic fermentation as a source of carotenoids (especially zeaxanthin).

The highest amounts of total chlorophylls were in not-fermented willow herb leaves and longer fermentation process decreased levels of chlorophylls (especially chlorophyll a).

The highest quantities of total sugars were found after 48 and 72 h anaerobic solid-state fermentation, but the highest content of sucrose was in not-fermented leaves. 

In summary, we can say that solid-state fermentation activates both the degradation of cell walls and the activity of microorganisms and enzymes, which leads to quantitative and qualitative changes in biologically active substances. Based on the data of this study, 72 h of anaerobic solid-state fermentation could be suggested for the food and pharmacy industry to produce willow herb leaf functional food products with higher carotenoids and sugar contents, and not-fermented leaves of willow herbs could be very useful for people due to high vitamin C and chlorophyll content.

## Figures and Tables

**Table 1:** The contents of vitamin C, DHA and LAA in willow herb leaves fermented under the different fermentation methods and duration.

Compounds/ Fermentation Duration	Vitamin C	DHA ^2^	LAA ^3^
	mg 100 g^−1^ DW ^4^		
Control (not-fermented)	658.06 ± 29.23 a ^1^	495.22 ± 25.72 a	162.83 ± 3.75 a
	Aerobic SSF ^5^ method		
SSF 24 h	413.77 ± 39.63 b	271.17 ± 39.45 b	142.59 ± 2.44 b
SSF 48 h	178.94 ± 7.24 cd	78.11 ± 15.15 c	100.83 ± 8.10 d
SSF 72 h	145.61 ± 7.56 d	66.53 ± 3.14 c	79.08 ± 10.25 e
	Anaerobic SSF method		
SSF 24 h	174.62 ± 12.85 cd	47.74 ± 4.73 c	126.88 ± 9.05 c
SSF 48 h	181.06 ± 1.04 c	53.84 ± 0.63 c	127.21 ± 0.81 c
SSF 72 h	200.67 ± 0.45 c	60.18 ± 2.06 c	140.50 ± 1.62 b
*p*-Value (SSF duration × SSF method)	<0.00001	<0.00001	<0.00001

^1^ Means within columns marked by different small letters are statistically significantly different at *p* < 0.05; ^2^ DHA—dehydroascorbic acid; ^3^ LAA—L-ascorbic acid; ^4^ DW—dry weight; ^5^ SSF—solid-state fermentation.

**Table 2:** The contents of total carotenoids and individual carotenoids in willow herb leaves fermented under the different fermentation methods and duration.

Compounds/ Fermentation Duration	Total Carotenoids	Zeaxanthin	Lutein	Beta-Carotene
	mg 100 g^−1^ DW ^2^			
Control (not-fermented)	124.52 ± 1.61 e ^1^	86.33 ± 0.91 c	30.61 ± 0.98 a	7.57 ± 0.07 a
	Aerobic SSF ^3^ method			
SSF 24 h	108.83 ± 0.99 f	87.22 ± 0.25 c	14.89 ± 0.75 c	6.72 ± 0.03 d
SSF 48 h	149.31 ± 1.16 ab	120.87 ± 1.17 a	21.16 ± 0.08 b	7.27 ± 0.08 b
SSF 72 h	151.51 ± 1.47 a	123.37 ± 1.64 a	20.61 ± 0.65 b	7.53 ± 0.09 a
	Anaerobic SSF method			
SSF 24 h	131.29 ± 3.95 d	111.44 ± 3.22 b	12.72 ± 0.66 d	7.13 ± 0.10 c
SSF 48 h	144.45 ± 2.32 c	123.72 ± 2.59 a	13.21 ± 0.35 d	7.51 ± 0.09 a
SSF 72 h	146.77 ± 2.60 bc	124.14 ± 2.53 a	15.09 ± 0.10 c	7.54 ± 0.02 a
*p*-Value (SSF duration × SSF method)	<0.00001	<0.00001	<0.00001	0.00124

^1^ Means within columns marked by different small letters are statistically significantly different at *p* < 0.05; ^2^ DW—dry weight; ^3^ SSF—solid-state fermentation.

**Table 3:** The contents of total chlorophylls, chlorophyll a and chlorophyll b in willow herb leaves fermented under the different fermentation methods and duration.

Compounds/ Fermentation Duration	Total Chlorophylls	Chlorophyll a	Chlorophyll b
	mg 100 g^−1^ DW ^2^		
Control (not-fermented)	371.47 ± 1.66 a ^1^	278.70 ± 0.25 a	92.76 ± 1.91 d
	Aerobic SSF ^3^ method		
SSF 24 h	321.36 ± 3.12 a	281.73 ± 2.53 a	39.62 ± 0.66 e
SSF 48 h	260.11 ± 17.05 b	151.65 ± 22.08 b	108.45 ± 5.39 c
SSF 72 h	201.70 ± 16.25 c	75.44 ± 16.25 c	126.26 ± 3.80 ab
	Anaerobic SSF method		
SSF 24 h	130.64 ± 4.38 d	34.84 ± 4.38 c	95.80 ± 3.93 d
SSF 48 h	156.80 ± 4.40 cd	39.51 ± 4.40 c	117.29 ± 1.33 bc
SSF 72 h	180.61 ± 0.44 cd	46.60 ± 0.44 c	134.02 ± 12.60 a
*p*-Value (SSF duration × SSF method)	0.00114	0.00013	<0.00001

^1^ Means within columns marked by different small letters are statistically significantly different at *p* < 0.05; ^2^ DW—dry weight; ^3^ SSF—solid-state fermentation.

**Table 4:** The contents of total sugars, fructose, glucose and sucrose in willow herb leaves fermented under the different fermentation methods and duration.

Compounds/ Fermentation Duration	Total Sugars	Fructose	Glucose	Sucrose
	mg 100 g^−1^ DW ^2^			
Control (not-fermented)	5.37 ± 0.20 c ^1^	1.08 ± 0.02 e	1.11 ± 0.04 cd	3.18 ± 0.17 a
	Aerobic SSF ^3^ method			
SSF 24 h	3.63 ± 0.04 d	1.37 ± 0.05 c	1.31 ± 0.02 c	0.96 ± 0.01 d
SSF 48 h	3.15 ± 0.04 e	1.19 ± 0.04 d	1.02 ± 0.01 d	0.94 ± 0.01 d
SSF 72 h	2.22 ± 0.01 f	0.62 ± 0.01 f	0.70 ± 0.01 e	0.91 ± 0.01 d
	Anaerobic SSF method			
SSF 24 h	5.91 ± 0.28 b	1.81 ± 0.05 b	2.50 ± 0.26 b	1.60 ± 0.03 b
SSF 48 h	6.84 ± 0.20 a	2.11 ± 0.05 a	3.25 ± 0.12 a	1.47 ± 0.04 c
SSF 72 h	6.63 ± 0.19 a	2.04 ± 0.07 a	3.14 ± 0.11 a	1.44 ± 0.02 c
*p*-Value (SSF duration × SSF method)	<0.00001	<0.00001	<0.00001	<0.00001

^1^ Means within columns marked by different small letters are statistically significantly different at *p* < 0.05; ^2^ DW—dry weight; ^3^ SSF—solid-state fermentation.

## Data Availability

Not applicable.
